# Progress in Cerebro-Spinal Surgery

**Published:** 1901-03-16

**Authors:** 


### Progress in Cerebro-Spinal Surgery.

In opening the discussion at the Congress of the Italian Surgical Society Prof. Biondi referred to his experience of some 47 spinal cases, and read notes of eight operations on the spine.[1] Of the eight cases one died five months afterwards from rupture of the spinal column, one relapsed, and six were cured of the disease which had produced disturbances of the cord and of the roots of the nerves; and in four of the six the nervous disturbances also were cured. Prof. Ceccherelli strongly denounced open operations for tuberculous affections of the spine. Prof. Durante referred to an interesting case in which he had removed the whole of one frontal lobe of the brain as far as the middle fossa of the skull together with the anterior portion of the temporal lobe. Prof. Roncali gave the histology of a sarcoma of the brain, in which he had found blastomycetes. From his experience he came to the following important conclusions:—(*a*) The blastomycetes cannot be accidental in malignant tumours. (*b*) When the blastomycetes assume the character of Russell's bodies, they cannot be cultivated. (*c*) They can always be found in malignant tumours with patience and adequate knowledge of their characters. (*d*) The so-called coccidia of malignant tumours are really blastomycetes, which are the real etiological factors of epithelioma and sarcoma.

**Surgery of Nerve-Ganglia.**—It may be of interest to review some of the recent experiences in this subject. At the Medical Society of London, Mr. J. Hutchinson, jun., read a paper on "Excision of the Gasserian Ganglion."[2] He had operated by trephening the temporal fossa and lifting up the dura mater and temporal lobe. In all his cases the neuralgia had been cured. Atrophy of the masticatory muscles, which necessarily resulted, did not give rise to marked inconvenience; but it is important to preserve the ophthalmic division of the fifth nerve to prevent anæsthetic ulceration of the cornea. He thought two or three years of pain should indicate the operation, also the affection of at least two branches of the fifth nerve.

It is only recently that the cervical sympathetic ganglia have been systematically removed. Thos. Jonnesco has recorded satisfactory results, giving a summary of 61 cases, which comprised glaucoma, Basedow's disease, and idiopathic epilepsy. In 43 of the cases total bilateral excision of the three cervical ganglia was carried out. No case was fatal. In Basedow's disease it is necessary to remember that the superior cervical ganglion gives nerve-fibres to the eyeball, and the inferior ganglion supplies the thyroid gland and accelerator fibres to the heart. Hence it is necessary to remove both the ganglia in that disease. In the case of epilepsy the effect is wrought by a vaso-dilatation of the cerebral arteries—of the carotid by removal of the superior ganglion, and of the vertebral by removal of the inferior ganglion. In ten cases of Basedow's disease six were cured and four improved. The disappearance of exophthalmos was immediate and the nervousness ceased next day. It is stated that of the cases of epilepsy six died subsequently of the disease or an intercurrent malady. This should be noted and its meaning sought.

Following on the lines of Jonnesco, Mr. Work Dodd has resected the superior cervical ganglion on both sides for glaucoma.[4] The factor utilised is that the removal of the ganglion produces a marked fall in the intra-ocular tension and strong contraction of the pupil, on the view that glaucoma is of central origin, not peripheral. In the operation the ganglion was sought for by opening the carotid sheath, separating the artery and vein, and retracting the vagus nerve forwards. The ganglion was then caught in forceps and twisted out so as to remove the nerve from as high as possible, and then the nerve was divided below. The pulse was somewhat affected towards the end of the operation. The report was too early to give the ultimate results.

Mr. Burghard has described three cases in which he removed the superior cervical ganglion.[5] The first operation was for sub-acute glaucoma. In this case the carotid sheath and its contents were unopened, but retracted well to the inner side. "Ten minutes after the patient's return to bed the face was markedly flushed, and the tension in both eyes was normal." Later the tension was T + 1; there was no retraction of the eyeball. "The operation had no beneficial effect whatever, while it actually caused the patient intense physical suffering for upwards of a fortnight."

The second case was one of severe pain following removal of part of the tongue for epithelioma. It was found that there was a secondary deposit in a cervical gland, which was matted to the superior cervical ganglion, both of which were removed in one mass. His intense pain never returned, and the pupil remained markedly contracted. The third case was one of tumour of the neck associated with excruciating pain, which, on dissection, proved to be a tumour of the superior and middle cervical ganglia about the size of a pigeon's egg, the removal of which completely cured the pain. It was a false neuroma. Burghard says the constant effects of the operation are ptosis, severe pain in the head on the side operated upon, congestion of the face, and contraction of the pupil.

**Surgical Treatment of Epilepsy.**—The surgical treatment of epilepsy is to-day in disrepute, reliable statistics only yielding 2 to 4 per cent. of cures according to Kocher.[6] The removal of bony fragments and cicatrices yields about 85 per cent. of cures. Kocher regards intracranial tension as a great cause of epilepsy and holds that the relief afforded by trephining is when the dura mater has been opened so as to make a yielding valve to compensate for variations in intracranial pressure.

**Musculo-spiral Paralysis from Fractured Humerus.**—The details of six cases have been recorded by W. W. Keen[7] with restoration of function in two of them.

*Case I.*—This was a child, aged 6, with T-shaped fracture into the elbow-joint with considerable deformity. At the operation, seven months afterwards, a fragment of the humerus was found drawing upon the nerve like a hook.

*Case II.* was an ununited fracture of the humerus with injury to the nerve and considerable laceration of the forearm. The operation was thirteen months after the accident and improvement was partial.

*Case III.* was five months after the accident and recovery was rapid and complete. In order to be able to approximate the ends of the nerve in this case over an inch of the humerus was resected.

*Case IV.* was one in which the nerve was sutured thirteen years after the accident. There was a considerable gap between the ends of the nerve which was accordingly bridged over by strands of catgut, but no improvement resulted.

*Case V.* ended in slight improvement, the operation having been performed nearly two years after the injury.

*Case VI.* was one in which the musculo-spiral nerve had been divided by a knife-blade, and the operative result was good. In this and in the previous case the operation consisted of the removal of a bulbous portion of the nerve at the site of injury, the continuity of the nerve not having been completely destroyed.

**Recurrent Luxation of the Ulnar Nerve.**—There is a valuable contribution to this subject by Dr. F. J. Cotton[8] giving three examples, their symptoms, and the literature of the subject. The first case, the result of a fall, left a swollen elbow and a tender cord in front of the inner condyle when the elbow was flexed, to be reduced again on extension of the elbow. By pad and splints the nerve was kept in position and in four months the patient was well, except for occasional darting pains. The second case, in a girl, had a similar course and presented like symptoms. Curiously enough, there was pain in the middle and ring fingers, not in the little finger as one would expect. By massage and splints much improvement was effected, but luxation still occurred after the conservative treatment. In the third case, that of a boy, the luxation was only partial, but there were local pain and tenderness.

Cotton divides luxation of the ulnar nerve into two classes (a) the *habitual* and (b) the *traumatic.* In the former class there is no known cause, and symptoms may or may not exist.

In the latter case symptoms are the rule. The injury need not be direct, habitual over-use of the elbow sufficing to cause luxation. The luxations are usually only temporary, produced by flexing the elbow. Permanent luxations are rare. The symptoms are tenderness, pain and numbness in the cutaneous distribution of the nerve, and some atrophy of the muscles supplied by it. Obviously there must be much tearing of the fibrous bridge over the nerve, and perhaps of part of the flexor corpi ulnaris muscle. Cases had been previously recorded by Blattmann (1851), Lutz (1879), and Zuckerkandl (1880). With adequate rest and support the traumatic cases got well, conservative treatment giving good results. In other cases operation may be required after trying palliative measures. This consists in trimming the fibrous covering of the nerve and stitching the edge of the triceps to the internal condyle over the nerve. In other cases a slip of fascia has been dissected up from the flexor group of muscles and stitched over the nerve to the triceps muscle.

[1] Brit. Med. Jour., Nov. 17, 1900. [2] Lancet, May 19th, 1900. [3] Epit. Brit Med. Jour., June 23rd, 1900. [4] Lancet, Oct. 13th, 1900. [5] Brit. Med. Jour.' Oct. 20th, 1900. [6] Ann. Surg., May, 1900. [7] Med. Chron., Aug., 1900' [8] Boston Med. and Sur. Jour., Aug. 2nd, 1900.

